# Modulation of temporal and occipital cortex by acupuncture in non-menstrual MWoA patients: a rest BOLD fMRI study

**DOI:** 10.1186/s12906-024-04349-w

**Published:** 2024-01-20

**Authors:** Yu-Chan Yang, Xiang-Yu Wei, Ying-Ying Zhang, Chun-Yang Xu, Jian-Ming Cheng, Zhi-Gang Gong, Hui Chen, Yan-Wen Huang, Jie Yuan, Hui-Hui Xu, Hui Wang, Song-Hua Zhan, Wen-Li Tan

**Affiliations:** 1 Department of Radiology, Shuguang Hospital, Shanghai University of Traditional Chinese Medicine, Shanghai, 201203 China; 2 Institute of Acupuncture and Anesthesia, Shuguang Hospital, Shanghai University of Traditional Chinese Medicine, Shanghai, 201203 China

**Keywords:** Blood-oxygen-level-dependent functional magnetic resonance imaging, Acupuncture, Non-menstrual migraine without aura, Low-frequency fluctuation, Degree centrality

## Abstract

**Objective:**

To investigate the changes in amplitude of low-frequency fluctuation (ALFF) and degree centrality (DC) values before and after acupuncture in young women with non-menstrual migraine without aura (MWoA) through rest blood-oxygen-level-dependent functional magnetic resonance imaging (BOLD fMRI).

**Methods:**

Patients with non-menstrual MWoA (Group 1, *n* = 50) and healthy controls (Group 2, *n* = 50) were recruited. fMRI was performed in Group 1 at 2 time points: before acupuncture (time point 1, TP1); and after the end of all acupuncture sessions (time point 2, TP2), and performed in Group 2 as a one-time scan. Patients in Group 1 were assessed with the Migraine Disability Assessment Questionnaire (MIDAS) and the Short-Form McGill Pain Questionnaire (SF-MPQ) at TP1 and TP2 after fMRI was performed. The ALFF and DC values were compared within Group 1 at two time points and between Group 1 and Group2. The correlation between ALFF and DC values with the statistical differences and the clinical scales scores were analyzed.

**Results:**

Brain activities increased in the left fusiform gyrus and right angular gyrus, left middle occipital gyrus, and bilateral prefrontal cortex and decreased in left inferior parietal lobule in Group 1, which had different ALFF values compared with Group 2 at TP1. The bilateral fusiform gyrus, bilateral inferior temporal gyrus and right middle temporal gyrus increased and right angular gyrus, right superior marginal gyrus, right inferior parietal lobule, right middle occipital gyrus, right superior frontal gyrus, right middle frontal gyrus, right anterior central gyrus, and right supplementary motor area decreased in activity in Group 1 had different DC values compared with Group 2 at TP1. ALFF and DC values of right inferior temporal gyrus, right fusiform gyrus and right middle temporal gyrus were decreased in Group1 at TP1 compared with TP2. ALFF values in the left middle occipital area were positively correlated with the pain degree at TP1 in Group1 (correlation coefficient r, *r* = 0.827, *r* = 0.343; *P* < 0.01, *P* = 0.015). The DC values of the right inferior temporal area were positively correlated with the pain degree at TP1 in Group 1 (*r* = 0.371; *P* = 0.008).

**Conclusion:**

Spontaneous brain activity and network changes in young women with non-menstrual MwoA were altered by acupuncture. The right temporal area may be an important target for acupuncture modulated brain function in young women with non-menstrual MwoA.

## Introduction

Migraine is a common type of primary headache with unclear etiology. Some studies suggest that migraine represents an overall dysfunction of multi-sensory integration, cognition and attention, emotional processing and executive function [1]. Epidemiological studies showed a global incidence of migraine to be approximately 12% [2], among which women make up the majority of migraine sufferers, with the highest incidence among women aged 18–49 [3]. The International Classification of Headache Disorders includes A1.1 the definitions of pure menstrual migraine without aura and menstrually related migraine without aura and non-menstrual migraine without aura. The diagnostic criteria of non-menstrual migraine without aura includes not fulfilling criterion to identify migraines that occur only or preferentially during the days around the menstruation [4]. The management of migraine had become a challenging global public health issue, and new treatment modalities are therefore mandatory. Studies had shown that acupuncture was safe and effective for migraine with its curative effect, simple procedure, low price and limited side effects [5], which thereby had been widely recognized and accepted in the treatment of migraine [6, 7].

Functional magnetic resonance imaging (fMRI) is a useful imaging modality that can reflect the changes of local functional activities within the human brain. Recent brain functional studies using fMRI had examined changes in brain structure and function associated with migraine [8, 9]. Based on the previous researches, migraine patients showed abnormal connectivity in the sensorimotor system, the resting default mode network (DMN), and the executive network [10, 11]. Some studies had shown that significant differences in intra-network connectivity in sensory and cognitive networks in adolescents with migraine aged 12–19 [12]. Also using ICA, some researchers had found both altered intra- and inter-network connectivity in brain networks involved in multisensory processing and cognitive control of pain in youth with migraine aged 9–17 [13]. Migraine patients exhibited abnormal thalamo-cortical dynamic functional network connectivity between the posterior thalamus and default mode and visual regions, also extended current findings regarding abnormal thalamo-cortical networks and dysrhythmia in migraine [14]. In another study, the left anterior central gyrus, left inferior parietal lobe, and left posterior central gyrus showed increased functional connectivity to the right frontoparietal network (rFPN) in migraine patients after standardized acupuncture treatment [15]. These results suggest that acupuncture altered the functional connectivity in the brain network and provided pain management in migraine patients. In an analysis of functional connectivity using the precuneus as the seed point, acupuncture treatment was reported to be able to relieve symptoms by enhancing cognitive ability [16]. Therefore, acupuncture may modulate the ascending input/descending pain modulation imbalance in the central nervous system in patients with migraine.

Previous studies have suggested that migraine in women are closely related to the menstrual cycle [17, 18]. There was evidence that acupuncture reduces the number of migraine days in patients with menstrual-related migraine compared to medication [19]. Few studies had reported changes in brain functional activity in non-menstrual young women with migraine without aura (MwoA) before and after acupuncture intervention [20]. Our previous study showed that acupuncture significantly regulated the functional connection between the insula subarea and other parts of the brain in this patient population [21]. However, the association between acupuncture treatment and resting brain activity, as well as the correlation between these potential functional activity changes and clinical symptoms, have not been illustrated in non-menstrual young women with MwoA. Therefore, we recruited non-menstrual young women with MwoA and analyzed the changes in brain activity at baseline and in the resting state after acupuncture intervention in this prospective study to explore the central nervous mechanism of acupuncture in migraine management.

## Materials and methods

### Patient recruitment

This clinical trial was approved by the Ethics Committee of Shuguang Hospital affiliated to Shanghai University of Traditional Chinese Medicine Clinical Trial Registry Platform, World Health Organization under registration number: 2019-766-121-01. A total of 50 patients with non-menstrual MWoA were enrolled in Group 1, and 50 health controls matched for age, gender, years of education and handedness with those in Group 1 were recruited as Group 2. All subjects were recruited at Shanghai University of Traditional Chinese Medicine between September 2021 and January 2022.

### Inclusion and exclusion criteria for migraine patients

Inclusion criteria: (1) right-handed, female patients aged between 18 and 35 years; (2) patients had the diagnosis of MwoA based on the International Classification of Headache Diseases III (ICHD-III) criteria; (3) patients did not receive any preventive medicine or acupuncture treatment for headache within the past 3 months; (4) the duration of migraine should be at least 6 months; (5) patients who had undergone headache episode at least once a month for the past 3 months and were not in the menstrual period when fMRI were performed; (6) patients who had signed the informed consent form. If any of the above criteria were not met, the patients were not enrolled in the study.

Exclusion criteria: (1) patients with alcohol or drug abuse; (2) patients with mental, neurological, cardiovascular, respiratory or renal disease, (3) patients with a history of head trauma of any other type of headache or loss of consciousness; (4) patients with contraindications to MRI such as claustrophobia; (5) patients with contraindications to acupuncture such as bleeding disorders.

### Inclusion and exclusion criteria for healthy controls

Inclusion criteria: right-handed female individuals who aged between 18 and 35 years and not in their menstruating period when undergoing fMRI scan were included.

Exclusion criteria: subjects with any type of primary or secondary headache, history of a clinically significant disorder, pregnancy or breast-feeding, contraindications to MRI, severe head deformity, or intracranial lesions were excluded.

### Study procedure

**Fig. 1:**
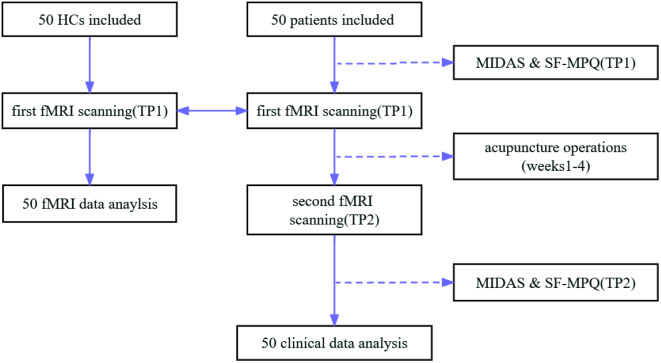
Flow chart showing the enrollment, treatment, and follow-up processes of the study. fMRI, functional magnetic resonance imaging; HCs: healthy controls. SF-MPQ: The Short-Form McGill Pain Questionnaire. MIDAS: The Migraine Disability Assessment Scale. TP1: Time Point 1, The migraine patients and healthy controls received first fMRI scans at baseline. At the same time, migraine patients received SF-MPQ & MIDAS assessment before acupuncture. TP2: Time Point 2, migraine patients received second fMRI scans and SF-MPQ & MIDAS assessment after the end of all acupuncture sessions

Patients in the Group 1 received resting-state functional magnetic resonance imaging (rs-fMRI) scan at baseline (time point 1, TP1) and at the end of all acupuncture treatments (time point 2, TP2). All patients were assessed for disease severity using the Migraine Disability Assessment Questionnaire (MIDAS) and the Short-Form McGill Pain Questionnaire (SF-MPQ) before and after acupuncture therapy. Subjects in Group 2 received only one rs-fMRI scan (at TP1) (Fig. 1).

### Acupuncture procedure

**Fig. 2:**
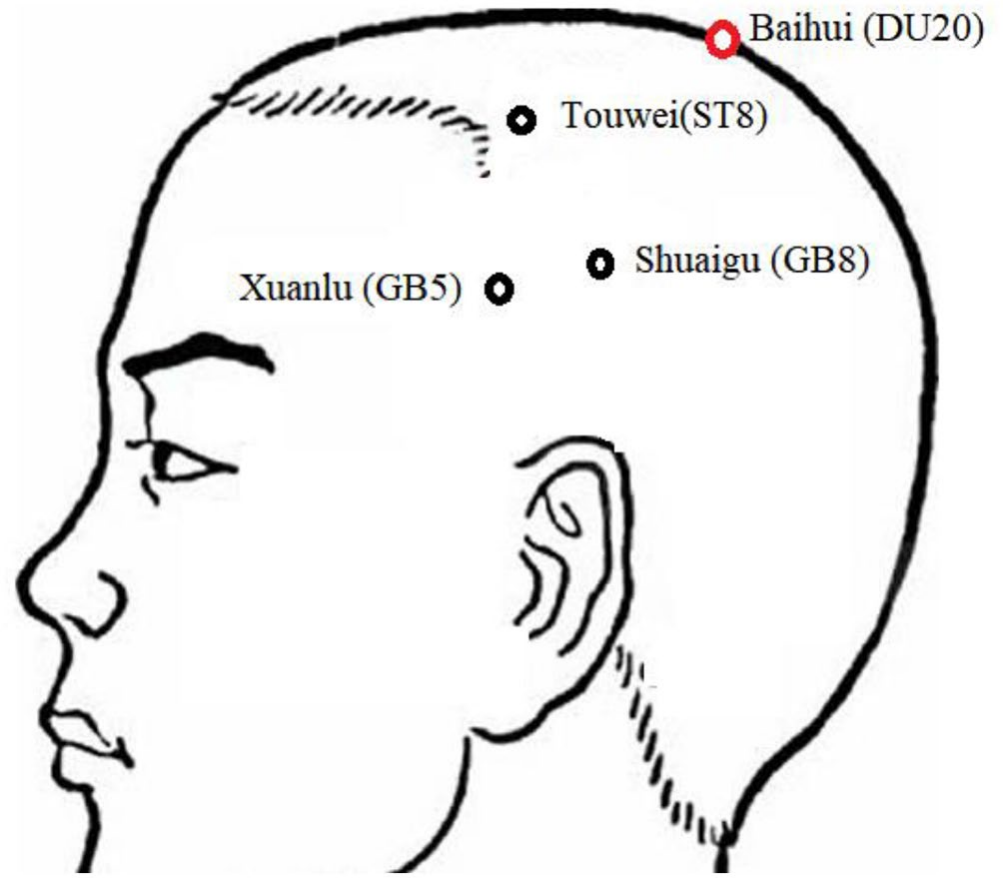
The location of four acupoints involved in this study: Baihui (DU20), Shuaigu (GB8), Xuanlu (GB5), Touwei (ST8).

All acupuncture procedures were performed by the same acupuncturist with over 3 years of experience. Acupoints were located according to the national standards for acupoint location (GB 12346-90). The following acupoints were selected: Baihui (DU20), Shuaigu (GB8), Xuanlu (GB5), and Touwei (ST8) (Fig. 2) according to the principle of main points + syndrome differentiation [22, 23]. DU20 and GB8 are the main points. GB5 and ST8 is the matching point. All patients received 8 sessions of acupuncture procedures for 4 weeks, with 2 sessions per week, each lasting 20 min. Disposable stainless steel acupuncture needles (0.35 × 40 mm, Huatuo brand) and electroacupuncture therapy instrument (SDZ-II, Suzhou Medical Supplies Factory Co., LTD.) were used for the procedures. Sterile single-use acupuncture needle with a length of 25–40 mm and a diameter of 0.25 mm were inserted with the purpose to achieve the Deqi sensation. Electric stimulation was applied at DU20 and GB8 on the affected side. The anode and cathode of each pair of electroacupuncture were respectively connected with the needle handle on the same side of the acupoint. The stimulation frequency was 2 Hz and with intensity varying from 0.1 to 1.0 mA until the patient felt comfortable. All patients agreed not to take any regular medications for migraine for the duration of the study. Subjects in Group 2 received no intervention.

### Rs-fMRI scanning

In order to ensure the consistency of study, fMRI scans were scheduled for all subjects after their menstrual cycle. Rs-MRI images were acquired by using a 3.0-Tesla scanner (uMR790 platform, United Imaging Medical Systems, Shanghai, China) with a 32-channel head coil. A sponge-built head holder was used to prevent head movements. The parameters were set as follows: (1) 3D-T1WI sequence structural imaging was performed with a T1-weighted magnetization-prepared rapid gradient echo (TR = 7.2 ms, TE = 3.1 ms, thickness = 1 mm, flip angle = 10°, FOV = 256 mm × 256 mm, 192 slices). (2) Functional images were acquired with a single-shot gradient recalled echo planar imaging (EPI) sequence (TR = 2000 ms, TE = 30 ms, thickness = 3.5 mm, flip angle = 90°, FOV = 224 mm × 224 mm, 33 slices, matrix = 64 × 64). Each scan lasted for 8 min, with a total of 240 time points. Subjects were instructed to relax with their eyes closed during the scanning process.

### Data processing

Image data were processed with MATLAB program (mathworks.com) and SPM 12 (https://fil.ion.ucl.ac.uk/spm) was used to preprocess the data, including the following main steps: (1) The first 10 volumes of each scan were removed to avoid instability due to T1-related relaxation effect. (2) Slice timing: time difference between data at each point in time was corrected and the head motion parameters of the subject in the scanning time series were obtained. (3) Realigning: the data at all time points were spatially aligned with the data collected at the first time point to obtain the head motion parameters of the subject in the scanning time series. (4) Co-registration and normalization: all collected data were resampled according to the Montreal Neurological Institute (MNI) standard template space with a 3 × 3 × 3 mm voxel size for spatial normalization. (5) Voxel-wise detrending: Nuisance regression was performed using the 24 head motion parameters, white matter, and cerebrospinal fluid signals as covariates. Linear trends were removed. (6) Filtering: the band-pass filtering range was set at 0.01–0.08 Hz to physiological level with high frequency noise. (7) smooth: a Gaussian kernel of 6 mm full width at half-maximum was used to smooth the images(this step should be after the calculation of DC but before ALFF).

Specific fMRI metrics can be used to characterize the cerebral regional and network characteristics in patient. Amplitude of low-frequency fluctuation (ALFF) was documented as a reflection of the spontaneous neural activity of the cerebral cortex. Degree centrality (DC) was the measurement of nodal influence across whole brain functional connections at the voxel level, which could identity the hubs in brain networks and provide functional connectivity of the entire brain. These two parameters were used in our study to reflect the cerebral regional and network characteristics in migraine patients.

ALFF and DC values were calculated using RESTplus (Resting-State fMRI Data Analysis Toolkit V1.8). ALFF value was yielded by transforming the square root from power and averaging from 0.01 to 0.08 Hz. The DC measure was computed based on a Pearson correlation coefficient with a cut-off of 0.25 (*p* = 0.001) and smoothed spatially with a Gaussian kernel of 6 mm full width at half-maximum (FWHM).

### Statistical analysis

The statistical software SPSS 20.0 was used for statistical analysis. Independent sample t-test was used to compare the difference of age in two groups. The Kruskal–Wallis H-test was used to assess differences in MIDAS and SF-MPQ scores between Group 1 at TP1 and Group 1 at TP2. The level of statistical significance was set at *P* < 0.05. The Mann–Whitney test was used to analyze the difference between Group 1 at TP1 and TP2. To avoid type I error, *P* < 0.025 was considered statistically significant based on a Bonferroni multiple comparison correction (0.05/2 = 0.025).

The MIDAS and SF-MPQ scores, as well as the change rates in MIDAS and SF-MPQ scores were calculated. The change rates in MIDAS and SF-MPQ scores were defined by the score difference between two time points divided by the scores from the initial time point.

Statistical analytic processes of ALFF and DC values were performed in RESTplus. The ALFF and DC values of two groups at baseline were compared by independent samples t-test with FDR correction (voxel-level correction, *P* < 0.001, Voxels > 60). The ALFF value and DC value of group 1 before and after EA were compared by paired T test with FDR correction (voxel-level correction, *P* < 0.001, Voxels > 20). The correlation between ALFF and DC values of brain regions with statistical difference, as well as MIDAS, SF-MPQ, MIDAS change rate (denoted as MIDAS change) and SF-MPQ score change rates (denoted as SF-MPQ change) in Group 1 were calculated by bivariate Pearson correlation analysis. *P* < 0.05 was considered statistically significant.

## Results

### Clinical data

The mean ages of subjects in Group 1 and Group 2 were 28.36 ± 3.61 and 27.60 ± 3.02 years, respectively (*P* = 0.26). Education level was transformed into the years of education and no significant difference in educational level was found between the two groups (*P* = 0.29) (Table 1). The MIDAS scores in Group 1 at TP2 were significantly lower than that at TP1 (at baseline) (*P* < 0.025, in pairwise comparisons). The SF-MPQ scores in Group 1 at TP2 were significantly lower than that at TP1 (at baseline) (*P* < 0.025, in pairwise comparisons) (Table 1).

**Table 1:** Demographic and clinical characteristics of participants

Data	Group 1 (*n* = 50)	Group 2 (*n* = 50)	P-value
Age (year)	28.36 ± 3.61 (18–33)	27.60 ± 3.02 (18–32)	0.26^a^
Education (year)	15.85 ± 2.72 (5–19)	16.63 ± 2.43 (5–19)	0.29^a^
Disease duration (year)	1.20 ± 8.19 (1–10)	NA	/
Frequency per month (times)	1.46 ± 2.55 (1–4)	NA	/
SF-MPQ score (TP1)	19.55 ± 7.55	NA	<0.025^b^
SF-MPQ score (TP2)	9.44 ± 4.85	NA	
SF-MPQ change	0.52 ± 0.35	NA	
MIDAS score (TP1)	14.28 ± 8.90	NA	<0.025^b^
MIDAS score (TP2)	6.50 ± 4.95	NA	
MIDAS change	0.54 ± 0.44	NA	

Data were expressed as mean ± SD (range). Group 1: Non-menstrual migraine patients. Group 2: healthy controls

NA = not applicable. Education: different types of education were transformed into the years of education: Primary school ≥ 5 years, Middle school ≥ 6 years, Undergraduate ≥ 4 years, Postgraduate ≥ 3 years. The disease duration of the migraine patients ranged from 1 year to 24 years. Frequency per month (times): The frequency of migraine attacks during the past 4 weeks. SF-MPQ: The Short-Form McGill Pain Questionnaire. MIDAS: The Migraine Disability Assessment Scale. TP1: Time Point 1, migraine patients and healthy controls received first fMRI scans at baseline. Meanwhile, migraine patients received SF-MPQ & MIDAS assessments before acupuncture. TP2: Time Point 2, migraine patients received second fMRI scans and SF-MPQ & MIDAS assessments after the end of all acupuncture sessions

^a^Independent-sample t test

^b^The SF-MPQ and MIDAS scores of Group 1 before and after acupuncture treatment was compared by paired t test

### fMRI data

**Fig. 3:**
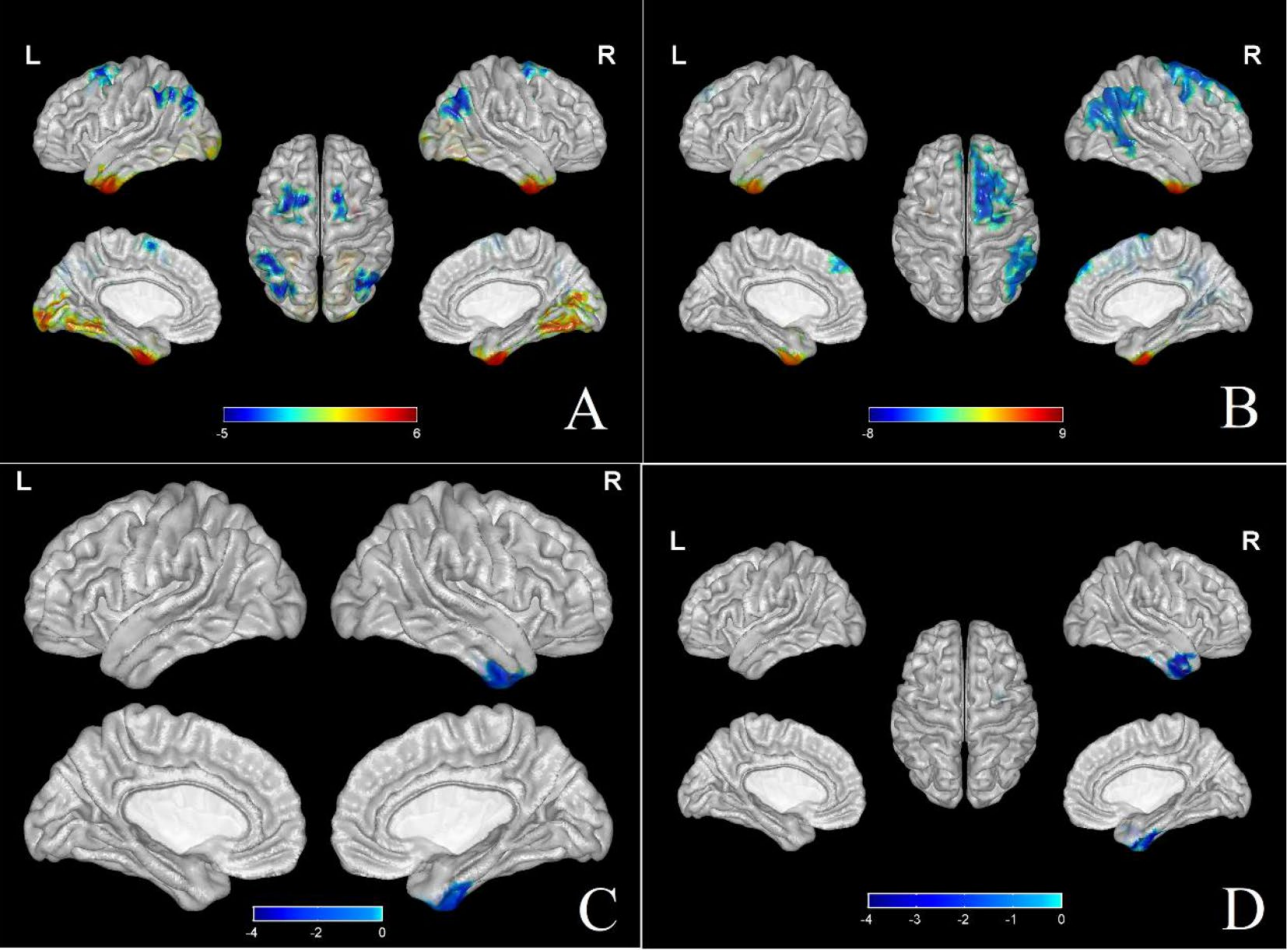
ALFF and DC values compared at different time points in 2 groups of patients. **A** Comparison of baseline ALFF values in various brain regions between patients with non-menstrual MWoA (Group 1) and healthy controls (Group 2). Increased ALFF values were observed in the left fusiform gyrus, while decreased ALFF values were observed in the right angular gyrus, left middle occipital gyrus, bilateral prefrontal cortex and left inferior parietal lobule. The brain regions with red-yellow color indicate a significantly increased ALFF value in Group 1 compared with Group 2, while the brain regions with green-blue color indicate a significantly decreased ALFF value in Group1 compared with Group 2 (*P* < 0.001; FDR corrected; Voxels > 60). **B** Comparison of brain regions with DC values at baseline between Group 1 and Group 2. Increased DC values were observed in bilateral fusiform gyrus, bilateral inferior temporal gyrus and right middle temporal gyrus. Decreased DC values were observed in right angular gyrus, right superior marginal gyrus, right inferior parietal lobule, right middle occipital gyrus, right superior frontal gyrus, right middle frontal gyrus, right anterior central gyrus and right complementary motor area. Brain regions with red-yellow color indicate a significantly increased DC value in Group 1 compared with Group 2, while brain regions with green-blue color indicate a significantly decreased DC value in Group1 compared with Group 2 (*P* < 0.001; FDR corrected; Voxels > 60). **C** Results in group 1: ALFF values of the right inferior temporal gyrus, right fusiform gyrus and right middle temporal gyrus after acupuncture significantly decreased compared with the time points before acupuncture (*P* < 0.001; FDR corrected; Voxels > 20). Brain regions with green-blue color indicate a significantly decreased ALFF value. **D** Results in group 1: DC values of the right inferior temporal gyrus, right fusiform gyrus and right middle temporal gyrus significantly decreased compared with the time points before acupuncture (*P* < 0.001; FDR corrected; Voxels > 20). Brain regions with green-blue color indicate a significantly decreased ALFF value

1. Baseline ALFF and DC values were comparable between Groups 1 and 2. Increased ALFF value was found in the left fusiform gyrus, while decreased ALFF values were identified in the right angular gyrus, left middle occipital gyrus, bilateral prefrontal cortex and left inferior parietal lobule. Brain regions with higher DC values were bilateral fusiform gyrus, bilateral inferior temporal gyrus and right middle temporal gyrus, reflecting the importance of network nodes in these regions. Brain regions with decreased DC values included the right angular gyrus, right superior marginal gyrus, right inferior parietal lobule, right middle occipital gyrus, right superior frontal gyrus, right middle frontal gyrus, right anterior central gyrus, and the right supplementary motor area (Table 2; Fig. 3A, B).

**Table 2:** Comparison of brain regions with ALFF and DC values at baseline between Group1 and Group 2

	Brain areas	MNI coordinates			Voxels	T
		X	Y	Z		
**ALFF**						
Group2 > Group1	Fusiform_L	−30	−6	−45	285	5.85
Group2 < Group1	Angular_R	36	−69	42	209	−4.69
Group2 < Group1	Occipital_Mid_L	−30	−78	36	121	−4.33
Group2 < Group1	Frontal_Sup_R	18	9	66	117	−4.32
Group2 < Group1	Frontal_Mid_L	−33	3	54	133	−4.29
Group2 < Group1	Parietal_Inf_L	−45	−46	47	228	−4.14
**DC**						
Group2 > Group1	Temporal_Inf_R	54	−30	−22	116	8.71
Group2 > Group1	Temporal_Inf_L	−50	−28	−20	139	8.71
Group2 > Group1	Temporal_Mid_R	57	−37	−3	229	8.71
Group2 > Group1	Fusiform_R	−30	−40	−25	81	8.71
Group2 > Group1	Fusiform_L	30	−6	−45	74	8.71
Group2 < Group1	Angular_R	46	−60	39	343	−5.42
Group2 < Group1	SupraMarginal_R	51	−33	42	229	−5.43
Group2 < Group1	Parietal_Inf_R	26	−59	62	160	−5.42
Group2 < Group1	Occipital_Mid_R	38	−80	−8	111	−5.42
Group2 < Group1	Frontal_Sup_R	22	30	41	490	−5.84
Group2 < Group1	Frontal_Mid_R	38	33	32	286	−5.84
Group2 < Group1	Precentral_R	40	−8	50	121	−5.84
Group2 < Group1	Supp_Motor_Area_R	8	50	−7	117	−5.84

ALFF: amplitude of low-frequency fluctuation; DC: Degree centrality; MNI: Montreal Neurological Institute; Group1: patients with migraine; Group2: Healthy control; R:right cerebral hemisphere; L: left cerebral hemisphere; *P* < 0.001; FDR corrected; Voxels > 60

2. Brain regions with ALFF and DC value changes in Group 1 before and after acupuncture intervention.

ALFF values of the right inferior temporal gyrus, right fusiform gyrus and right middle temporal gyrus decreased after acupuncture intervention. Consistently, DC values of the right inferior temporal gyrus, right fusiform gyrus and right middle temporal gyrus decreased after acupuncture (Table 3; Fig. 3C, D).

**Table 3:** Comparison of ALFF and DC values before and after acupuncture in Group 1

	Brain areas	MNI coordinates			Voxels	T
		X	Y	Z		
**ALFF**						
TP2 < TP1	Temporal_Inf_R	44	7	−39	134	−2.92
TP2 < TP1	Fusiform_R	36	−9	−42	68	−3.27
TP2 < TP1	Temporal_Pole_Mid_R	44	15	−32	22	−3.09
**DC**						
TP2 < TP1	Temporal_Inf_R	42	3	−42	145	−3.89
TP2 < TP1	Fusiform_R	38	−10	−41	81	−3.68
TP2 < TP1	Temporal_Pole_Mid_R	42	16	−33	57	−3.53

ALFF: amplitude of low-frequency fluctuation; DC: Degree centrality; MNI: Montreal Neurological Institute; TP2: after the acupuncture treatment; TP1: before the acupuncture treatment; R:right cerebral hemisphere; L: left cerebral hemisphere; *P* < 0.001; FDR corrected; Voxels > 20

### Correlation analysis between ALFF or DC values and SF-MPQ or MIDAS scores

**Fig. 4:**
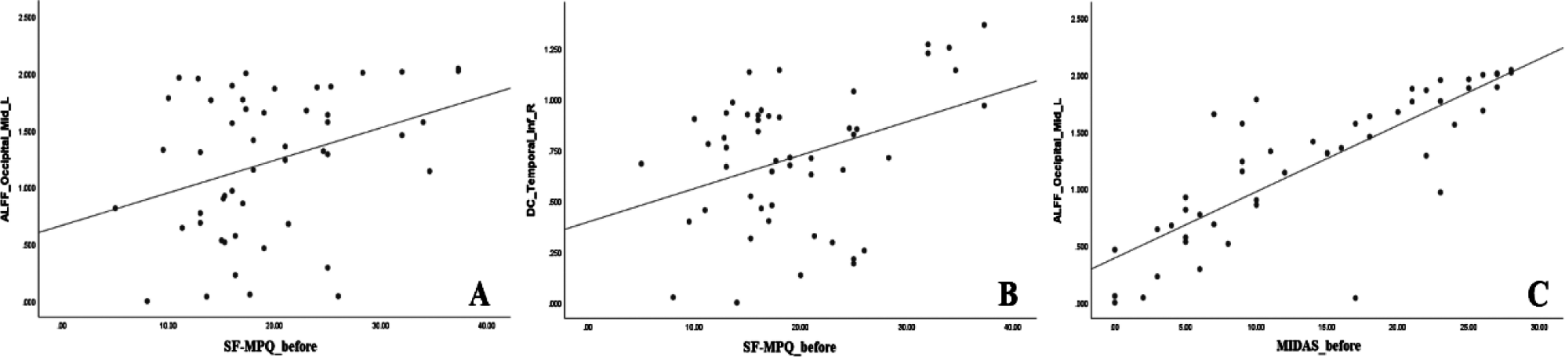
Correlation analysis between ALFF or DC values and SF-MPQ or MIDAS scores. **A** SF-MPQ scores were positively correlated with ALFF values of the left middle occipital area at TP1 in Group1 (*P* = 0.015, *r* = 0.343). **B** SF-MPQ scores was positively correlated with DC values of right inferior Temporal at TP1 in Group 1 (*P* = 0.008, *r* = 0.371). **C** MIDAS scores were positively correlated with ALFF values of the left middle occipital area before acupuncture (time point 1, TP1) in Group 1 (*P* < 0.01, *r* = 0.827)

The SF-MPQ score was also positively correlated with the ALFF value of the left middle occipital area at TP1 in Group 1 (*P* = 0.015, *r* = 0.343). Meanwhile, the SF-MPQ scores was positively correlated with DC values of the right inferior temporal area at TP1 in Group 1 (*P* = 0.008, *r* = 0.371). The MIDAS score was positively correlated with the ALFF value of the left middle occipital area at TP1 in Group 1 (*P* = 0.0036, *r* = 0.827) (Fig. 4).

## Discussion

### Characteristics of changes of brain spontaneous activity and brain network in young women with non-menstrual MwoA

The left fusiform gyrus was associated with increased ALFF value in MwoA patients compared with healthy subjects in this study. Decreased ALFF values were detected in the right angular gyrus, left middle occipital gyrus, bilateral prefrontal cortex and left inferior parietal lobule. Brain regions with higher DC values, including bilateral fusiform gyrus, bilateral inferior temporal gyrus and the right middle temporal gyrus, reflected the importance of network nodes. Brain regions with decreased DC values included the right angular gyrus, right superior marginal gyrus, right inferior parietal lobule, right middle occipital gyrus, right superior frontal gyrus, right middle frontal gyrus, right anterior central gyrus, and the right supplementary motor area. ALFF measures the intensity of spontaneous low-frequency fluctuations in the blood-oxygen-level-dependent (BOLD) signal, typically in the frequency range of 0.01 to 0.1 Hz [24]. ALFF is thought to reflect regional neural activity and has been associated with the baseline metabolic activity of the brain [25]. ALFF has been used as a marker for baseline neural activity and has been associated with various cognitive and sensory processes [26]. ALFF provides valuable information about the intensity of neural activity in a given region. DC measures the number of connections a node (brain region) has with other nodes in a functional brain network [27]. Higher DC values suggest that a particular region is more central or functionally connected within the network. Changes in DC can indicate alterations in the strength or pattern of connections between brain regions, providing insights into how acupuncture may influence the broader network dynamics [28]. ALFF and DC provide additional information about local and network-level changes in brain activity [29, 30]. In our study, young women with non-menstrual MwoA showed decreased baseline spontaneous activity and brain network disturbances in the default mode network (DMN) of the prefrontal cortex, angular gyrus, superior frontal gyrus, middle frontal gyrus, inferior parietal lobule, and the inferior temporal gyrus. The DMN is activated when the brain is awake at rest and inhibited when focusing on external tasks, which is involved in self-awareness, episodic memory, and ongoing cognitive and emotional activities [31]. Recent studies have demonstrated that DMN is the primary network affected by chronic pain and plays an important role in pain regulation in migraines [32]. Consistently in our study, DMN functions responsible for sensory integration, pain cognition and attention regulation were decreased in young women with non-menstrual MwoA. The prefrontal cortex in DMN was involved in complex cognitive behavior plannings [33, 34]. In our study, we found decreased DC values in the prefrontal cortex, superior frontal gyrus, and middle frontal gyrus that may indicate some disruption in the prefrontal lobes associated with pain processing. The sensorimotor circuit projects from the primary sensorimotor cortices such as the premotor area (PMA), frontal lobe, precentral gyrus, posterior central gyrus, SMA and relevant cortices to the putamen. Feedback signals would then return to these areas via the thalamus. Sensorimotor circuits were reported to be involved in affective motivation, cognitive assessment, and pain memory [35]. The anterior central gyrus was part of the sensorimotor cortex and was involved in pain management [36]. Brain imaging studies on pain induction suggested that the primary somatosensory (S1), secondary somatosensory (S2), insula, prefrontal cortices and other brain regions could be regarded “pain matrix”, whose functions were abnormal in the process of pain induction [37], which was consistent with our results. Dysfunctions of DMN and most regions of the sensorimotor circuit were involved in the neural mechanism of non-menstrual MwoA in female [38]. In this study, we found that DMN and sensorimotor circuit had a cross effect on the regulation and processing of pain, which could affect sensory, emotional and cognitive processing at multiple levels.

The occipital cortex was the hub for visual perception and processing, and the temporo-occipital cortex also had complex functional connections including multisensory integration responsible for visual, auditory, and tactile information [39, 40]. The indicator of occipital activity and neural synchronous were lower in migraine patients, and were generally thought to be associated with visual aura [41, 42]. We also observed functional changes in the occipital cortex in young women with migraine without aura. In addition, MIDAS and SF-MPQ scores were positively correlated with ALFF values in the left middle occipital gyrus at TP1 in Group 1, and SF-MPQ scores were positively correlated with DC values in the right inferior temporal gyrus at TP1 in Group 1. We speculated that photophobia or hypersensitivity to light during migraine episodes might be related to visual and auditory disturbances caused by abnormal spontaneous activity of the occipital cortex, which was consistent with the reports [43, 44]. There was a study demonstrated occipital nerve stimulation to be a potentially effective treatment for chronic migraine [44].

### The right temporal area serves as an important target of acupuncture brain function modulation in young women with non-menstrual MwoA

ALFF and DC values showed synchronous decreases in patients with non-menstrual MwoA after acupuncture intervention, and the relevant brain regions were located in the right inferior temporal gyrus, right fusiform gyrus and right middle temporal gyrus. This suggests a synchronization of local spontaneous activity and brain network in these patients and demonstrated that the right inferior temporal gyrus, middle temporal gyrus and fusiform gyrus played important roles in the pathogenesis of non-menstrual MwoA. The temporal lobe is reported to be involved in a variety of functional processes related to sensory processing, memory and language ability [44]. In this study, ALFF and DC values of the right inferior temporal gyrus, middle temporal gyrus and fusiform gyrus decreased in young women with non-menstrual MwoA after acupuncture, reversing its increasing trend in migraine without intervention. Migraine was an comprehensive disorder with multisensory integration including cognition and attention, emotional processing, and executive function [45]. The temporal lobe was considered to be an important nodal pathway for ascending and descending pain regulatory signals [46]. Functional and structural change in the temporal lobe may trigger a wide range of migraine symptoms, such as pain and cognitive disorders. The fusiform gyrus was an important part of visual identification network, and was also the processing area for auxiliary visual perception. In young women with non-menstrual MwoA of spontaneous brain activity and brain network abnormality, our results revealed the changes in the visual cortex and proved that the fusiform gyrus was associated with pain perception and cognitive processing. Moreover, spontaneous brain activity and corresponding brain network changes were observed in young women with non-menstrual MwoA after acupuncture intervention. The affected brain regions were located in a component of the right temporal lobe involved in higher visual processing, especially in object recognition and category recognition [47]. Meanwhile, we found in young women with non-menstrual MwoA that brain regions that are closely related to pain processing, such as the prefrontal cortex, insular cortex and anterior cingulate cortex, were at “low threshold”, while visual and auditory information, negative emotion and memory functions in the temporal lobe were abnormally activated, which can cause pain transmission,. Acupuncture may modulate pain by “suppressing” abnormal activation in the “low threshold” temporal lobe region and by reducing the spontaneous activity of temporal lobe and the importance of network node in migraine episodes [48]. Changes of spontaneous activity pattern in the fusiform gyrus, middle temporal gyrus and inferior temporal gyrus are involved in pain perception, pain processing and multisensory integration, which serve an important mechanism of acupuncture in alleviating migraine. Based on these findings, we speculate that the right temporal lobe may play a key role in the onset of non-menstrual MwoA in young women.

In this study, we used the brain imaging studies to identify the pain modulation-related key regions of acupuncture, including DMN. The current study aimed to examine the effects of acupuncture in regulating brain connectivity of the abovementioned pain modulation-related key regions in migraine patients. Recent evidence suggested that DMN can be divided into different subnetwork systems that underlie different cognitive processes [49, 50]. The temporal parietal junction subnetwork is involved in self-processing and social cognition [51]. ALFF and DC values of the non-menstrual MwoA decreased after acupuncture intervention, and it further revealed that acupuncture intervention for non-menstrual MwoA could restore the integrity of DMN. Multiple regions in the DMN could act as brain network hubs and play an important role in integration of brain information and migraine pathophysiology [52, 53]. In particular, the temporal parietal junction subnetwork played a greater role in regulating pain emotion. Evidence suggests that chronic pain could not only impaire subjects’ ability to control pain, but also alter cognitive and emotional regulations of pain, leading to higher pain intensity or longer pain duration [54, 55]. We speculate that the effects of acupuncture therapy may reverse the brain changes associated with chronic pain and gradually modulate the cognitive and emotional aspects of pain. The fusiform gyrus constitutes a pivotal component of the visual recognition network, playing a crucial role in sophisticated visual processing, particularly in the realms of object recognition and category identification [48]. Serving as a supplementary region in visual processing, the inherent functional activity pattern of the fusiform gyrus underwent alterations in patients with non-menstrual MWoA following acupuncture intervention. Moreover, previous studies have implicated the fusiform gyrus in pain perception and cognitive processing [56]. This observation could elucidate the perturbation in functional activity within the fusiform gyrus discerned in diverse pain disorders [57, 58]. In this study, decreased intrinsic connectivity within the fusiform gyrus in non-menstrual MWoA might provide a neuroanatomical basis for interpreting the real acupuncture mechanism. This study found that the consistently greater magnitudes shown in TP1 compared to TP2 for the analysis of static brain activity. It could be attributed to natural variations in baseline brain activity. Without an intervention, natural variability in brain activity could result in greater differences between baseline measurements. Moreover, it could be attributed to the cumulative effects over the entire course of acupuncture sessions at TP2. The immediate response to acupuncture may be more pronounced, and as the sessions progress, the cumulative effects may stabilize or reach a plateau.

There were some limitations in this study. First, the sample size was small and all subjects were female college students with relatively mild symptoms. Therefore, caution should be taken in generalizing our findings to very severe migraine patients. Second, the subjects enrolled were young women with non-menstrual MwoA, while male migraine patients, women younger than 16 years or older than 35 years of age with non-menstrual MwoA, and women with menorrhagia associated migraine were excluded. Therefore, it is necessary in future studies to optimize the design by increasing the sample size, expanding the inclusion criteria, and adding patient factors such as age and gender for subgroup analysis. Due to the strict inclusion criteria and specific intervention protocol, the results of this study should be applied with caution in migraine patients and patients undergoing acupuncture. The future studies recommend the implementation of longitudinal studies to assess the sustained effects of acupuncture over time. This could involve tracking changes in brain activity and clinical outcomes beyond the immediate post-acupuncture period to evaluate the durability of the observed modulations of acupuncture.

## Conclusion

Abnormal brain activity in certain brain regions were identified in young women with non-menstrual MwoA. Acupuncture intervention affects the sensory, emotional, memory, visual and auditory perceptions and processing areas in this patient population. ALFF and DC values of the right temporal lobe may serve as a potential imaging biomarker to predict the analgesic effect of acupuncture in young women with non-menstrual MwoA.

## Data Availability

The datasets used and/or analyzed during the current study are available from the corresponding author on reasonable request.
